# Clinical features of audible upper airway secretions (“death rattle”) in patients with cancer in the last days of life

**DOI:** 10.1007/s00520-024-08634-9

**Published:** 2024-06-12

**Authors:** Andrew Davies, Melanie Waghorn, Simon Skene

**Affiliations:** 1 University of Surrey, Guildford, UK; 2 Trinity College Dublin, University College Dublin, Our Lady’s Hospice, Dublin, Ireland; 3 Surrey Clinical Trials Unit, University of Surrey, Guildford, UK; 4 Education & Research Centre, Our Lady’s Hospice Dublin, Harold’s Cross, Dublin, D6W RY72 Ireland

**Keywords:** Palliative care, terminal care, respiratory sounds, death rattle

## Abstract

**Purpose:**

Audible upper airway secretions (“death rattle”) is a common problem in cancer patients at the end-of-life. However, there is little information about its clinical features.

**Methods:**

This is a secondary analysis of a cluster randomised trial of clinically-assisted hydration in cancer patients in the last days of life. Patients were assessed 4 hourly for end-of-life problems (including audible secretions), which were recorded as present or absent, excepting restlessness/agitation, which was scored using the modified Richmond Agitation and Sedation Scale. Patients were followed up until death.

**Results:**

200 patients were recruited, and 186 patients died during the study period. Overall, 54.5% patients developed audible secretions at some point during the study, but only 34.5% patients had audible secretions at the time of death. The prevalence of audible secretions increased the closer to death, with a marked increase in the last 12–16 h of life (i.e. the prevalence of audible secretions was highest at the time of death). Of those with audible secretions at the time of death, 24 had had a previous episode that had resolved. Development of audible secretions was not associated with use of clinically-assisted hydration, but there was an association between audible secretions and restlessness/agitation, and audible secretions and pain. However, most patients with audible secretions were not restless/agitated, or in pain, when assessed.

**Conclusion:**

Audible secretions (“death rattle”) are common in cancer patients at the end-of-life, but their natural history is extremely variable, with some patients experiencing multiple episodes during the terminal phase (although not necessarily experiencing an episode at the time of death).

## Introduction

Achieving a “good death” is the primary objective of end-of-life care (palliative care), although what constitutes a good death is highly personal, and studies suggest certain discrepancies between patients, their families, and their healthcare professionals [1–3]. Nevertheless, most research studies report that control of pain and symptoms is the foremost priority for both patients and their families (and equally for healthcare professionals involved in end-of-life care) [1, 2, 4].

Although a sign rather than a symptom, audible upper airway secretions (a.k.a. “death rattle”, “noisy breathing”, “terminal secretions”) is one of the most common problems encountered in patients at the end-of-life [5, 6]. Thus, the reported prevalence is 12–92% [7]. Importantly, it is often a cause of significant distress to family members and healthcare professionals [8, 9], although apparently not to patients (who are often semiconscious / unconscious when they develop this problem) [5, 10].

The “rattle” relates to turbulent airflow around / through the upper airway secretions, and is affected by the volume of secretions, the airways resistance (calibre of airways), and the rate of respiration [11]. Bennett proposed two distinct subtypes of audible upper airways secretions: a) type 1 – due to accumulation of saliva: these secretions are more likely to respond to anticholinergic medication, although such treatment does not remove secretions already present; b) type 2 – due to accumulation of bronchial secretions: these secretions are inherently less likely to respond to anticholinergic medication [11]. Impaired swallowing, impaired cough reflex, and supine / semi-recumbent positioning result in the pooling of these secretions in the oropharynx and bronchi respectively [11].

Despite being a common / important clinical problem there is a dearth of information about its clinical features, and particularly its association with other end-of-life problems (e.g. pain, terminal agitation). Hence, we decided to review the relevant prospective observations obtained from our feasibility study of clinically assisted hydration (CAH) in cancer patients in the last days of life (“CHELsea I study”) [12]: this data was not analysed in the original publication, as the information was not pertinent to the principal outcomes of the study.

## Methodology

The CHELsea I study was a cluster randomised trial of CAH in cancer patients in the last days of life [12]. The study was funded by the Research for Patient Benefit programme of the National Institute of Health Research (grant / award number ‘PB-PG-0613–31100’), sponsored by the University of Surrey, supported by the Surrey Clinical Trials Unit, and ethically approved by the London—Bromley Research Ethics Committee (14/LO/1543; 03/10/2014). It was conducted in accordance with the Declaration of Helsinki. The study was registered on ClinicalTrials.gov (reference NCT02344927; 26/01/2015).

The study was undertaken at four cancer centres, and eight hospices, in the United Kingdom. The sample size (200 participants) was based on a recommendation for sample sizes for feasibility studies involving a cluster randomised methodology [13]: the number of clusters (12 study sites) was based on the required sample size, and the proposed study duration (i.e. 1 year). As discussed, this was a feasibility study, and recruitment was one of the predetermined main criteria for success (i.e. 200 participants in 1 year).

The study sites were randomised to one of two “standard” (typical) interventions: arm ‘A’ involved continuance of / support with oral intake, regular mouth care, and usual management of pain and symptoms; arm ‘B’ involved continuance of / support with oral intake, regular mouth care, usual management of pain and symptoms, and CAH. Fluids were administered either intravenously or subcutaneously, and the fluid used was dextrose saline, and the volume of fluid used was based upon the patient’s weight.

All patients within study sites were eligible for inclusion in the study, assuming they met all of the inclusion criteria (and did not meet any of the exclusion criteria). The inclusion criteria were: a) diagnosis of cancer; b) age ≥ 18 yr; c) estimated prognosis ≤ 1 week (MDT opinion); and d) patient unable to maintain sufficient oral intake (1 L/day). The exclusion criteria were: a) patient is dehydrated; b) patient has hyperactive delirium / “terminal agitation” (primary objective of definitive study); c) relevant advance directive to refuse treatment; d) indication for CAH; e) contra-indication to CAH; f) contra-indication to peripheral cannulation; g) CAH or clinically-assisted nutrition already being administered; and h) patient is likely to be transferred to another setting.

Consent was sought from the patient whenever possible, or advice from a ‘personal consultee’ when the patient was unable to provide consent, or advice from a ‘nominated consultee’ when the patient was unable to provide consent and there was no personal consultee (as per the United Kingdom’s Mental Capacity Act 2005) [14]. In this study, the personal consultee was a relation or a friend of the patient, and the nominated consultee was the site Study Guardian (i.e. a senior healthcare professional independent of the research team). Moreover, in patients that were initially able to consent and that were subsequently deemed to have lost capacity, a personal/nominated consultee was required to confirm continued involvement in the study.

Patients were assessed every 4 h for relevant end-of-life problems (including pain, agitation / restlessness, audible upper respiratory secretions), which were recorded as either present or absent, with the exception of restlessness / agitation, which was scored using the modified Richmond Agitation and Sedation Scale (m-RASS) [15, 16]. All assessments were undertaken by the clinical team, as were decisions to manage relevant end-of-life care problems. End-of-life care problems were managed according to local protocols (“usual” clinical practice at the study sites), and the indications for / scheduling of all medications were recorded. Patients were followed up until death, or for a maximum of 14 days (end of study).

The full data set from the CHELsea I study was utilised in this post-hoc analysis. An m-RASS score of > 0 was deemed to represent the presence of agitation. Descriptive statistics are used to report most outcomes (e.g. numbers and percentages; median and range). Standard statistical tests were used to determine associations between patient characteristics and other end-of-life problems and the development of audible upper respiratory secretions (i.e. Chi-squared tests, Mann Whitney U tests). Odds ratios were used to assess the relationship between the presence of agitation or pain and the presence of audible upper respiratory secretions.

## Results

Two hundred patients were recruited to the study, 199 (99.5%) participants completed the study, and 186 (93%) patients died during the study period (see Table 1). [One patient was withdrawn due to an improvement in their condition]. The median survival of the deceased patients was three days (range < one day to 13 days). In total, 4739 sets of 4 hourly observations were undertaken (i.e. 93.5% potential observations), and the median number of observations per participant was 17 (range 0 to 82).

**Table 1:** Study population (patients with data that died during study)

Characteristic	Number of subjects (*n* = 182)
Age	
Median	73 yr
(range)	(28–98 yr)
Sex	
Female	105 (58%)
Male	77 (42%)
Ethnicity	
Asian/Asian British	1 (0.5%)
Black/Black British	1 (0.5%)
White	168 (92.5%)
Mixed	2 (1.0%)
Not stated	10 (5.5%)
Cancer diagnosis	
Breast	15 (8.0%)
Endocrine	5 (3.0%)
Gastrointestinal	60 (33.0%)
Gynaecology	16 (9.0%)
Haematology	11 (6.0%)
Head & neck	4 (2.0%)
Lung	25 (13.5%)
Neurology	7 (4.0%)
Skin	8 (4.5%)
Unknown	10 (5.5%)
Urology	15 (8.0%)
Other	6 (3.5%)
Co-morbidities	
Cardiac	62 (34.0%)
Gastrointestinal	32 (17.5%)
Neurological	29 (16.0%)
Renal	19 (10.5%)
Respiratory	35 (19.0%)

**Fig. 1:**
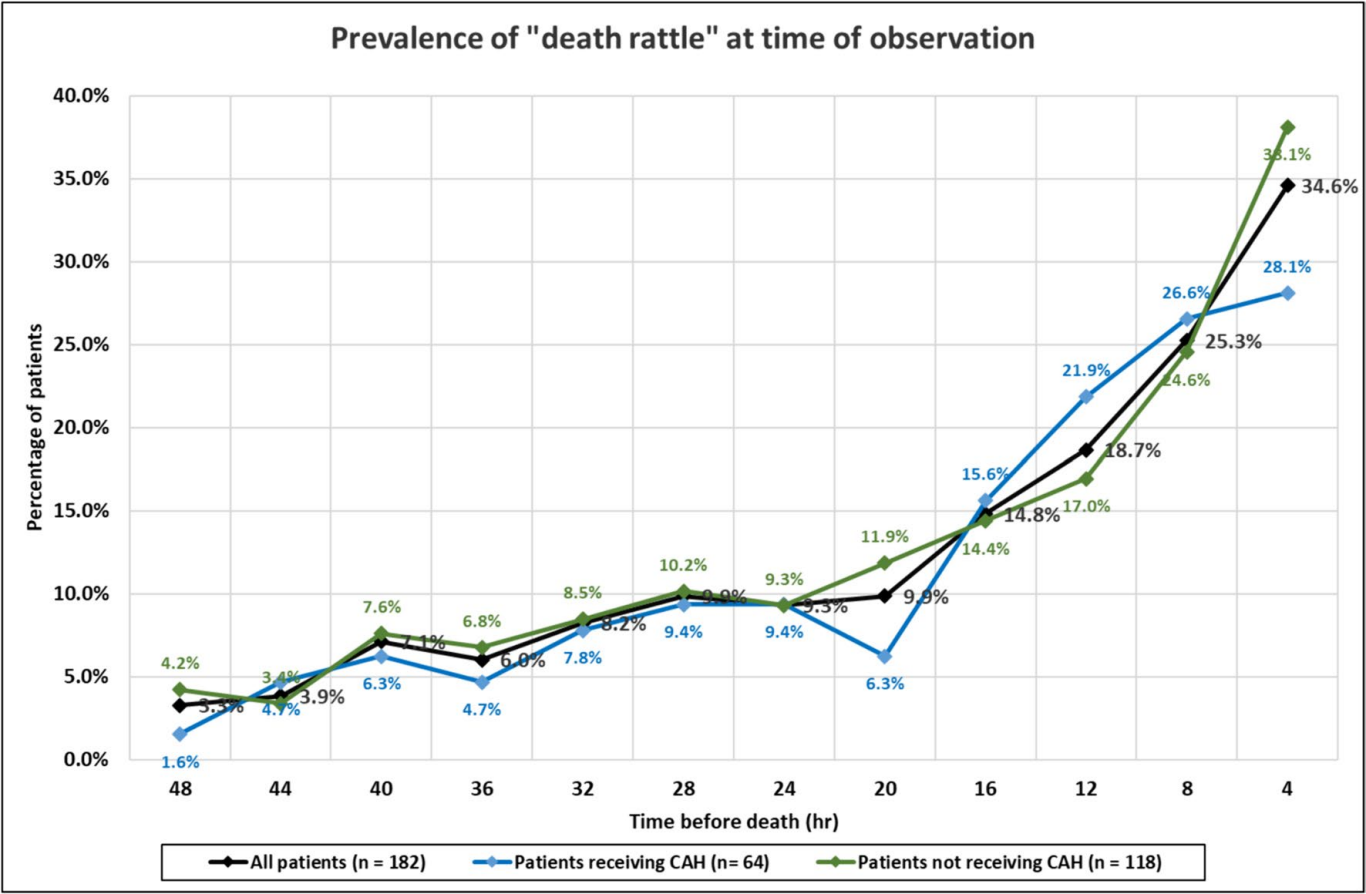
Prevalence of audible upper airway secretions (“death rattle”) in last 48 h of life

Of the deceased patients with data (*n* = 182), 99 (54.5%) patients developed audible upper airway secretions (“death rattle”) at some point during the study, but only 63 (34.5%) patients had them at the time of death. However, the 4 hourly prevalence of audible secretions increased the closer to death, with a marked increase in the last 12–16 h of life (Fig. 1). In other words, the prevalence of audible secretions was highest at the time of death. Of the patients with audible secretions at the time of death, 25 had had a previous episode that had resolved during the study period: 18 patients had a single episode, five had two episodes, and two had three episodes. The median duration of these episodes was < 4 h (with a range of < 4 h to < 32 h). [< 4 h means that the episode had resolved by the next nursing observation (and so on)].

As implied, 36 patients had audible secretions at some point during the study but not at the time of death: 20 patients had a single episode, four had two episodes, five had three episodes, six had 4 episodes, and one had five episodes. The median duration of these episodes was again < 4 h (with a range of < 4 h to < 32 h). Many of these episodes resolved spontaneously, and relatively few responded to as-required anticholinergic medication when utilised (see Table 2).

**Table 2:** Relationship between medication utilisation and resolution of episodes of audible upper airway secretions

Patients with no audible secretions at time of death (*n* = 36)	
Outcome of resolved episodes	Number of episodes (*n* = 72)
Spontaneous improvement (no medication given)	39
Improvement following as-required dose of anticholinergic medication	18
No improvement following as-required dose(s) of anticholinergic medication	13
No data to determine efficacy of anticholinergic medication	2
Patients with audible secretions at time of death (*n* = 25)	
Outcome of resolved episodes	Number of episodes (*n* = 34)
Spontaneous improvement (no medication given)	18
Improvement following as-required dose of anticholinergic medication	8
No improvement following as-required dose(s) of anticholinergic medication	8

The development of audible secretions at any point was not associated with age (Mann Whitney U test: *p* = 0.3408), cancer diagnosis (χ^2^ test: *p* = 0.1752), co-morbid respiratory disease (χ^2^ test: *p* = 0.2634), co-morbid cardiovascular disease (χ^2^ test: *p* = 0.8198), co-morbid renal disease (χ^2^ test: *p* = 0.4178), or co-morbid neurological disease (χ^2^ test: *p* = 0.7527). However, it was associated with male sex [χ^2^ (1, *n* = 182) = 5.9764, *p* = 0.0145]. Interestingly, there was no such association when considering patients with audible secretions at the time of death [χ^2^ (1, *n* = 182) = 1.8787, *p* = 0.1705].

The proportion of participants that developed audible secretions at any point during the study was similar in the two study groups, i.e. 54.5% in the group receiving CAH, and 54.0% in the group not receiving CAH [χ^2^ (1, *n* = 182) = 0.0034, *p* = 0.9536]. Likewise, the proportion of participants that had audible secretions at death was not dissimilar in the two study groups, i.e. 28.0% in the group receiving CAH, and 38.0% in the group not receiving CAH [χ^2^ (1, *n* = 182) = 1.8372, *p* = 0.175275]. The 4 hourly prevalence of audible secretions increased the closer to death in both groups (Fig. 1).

**Fig. 2:**
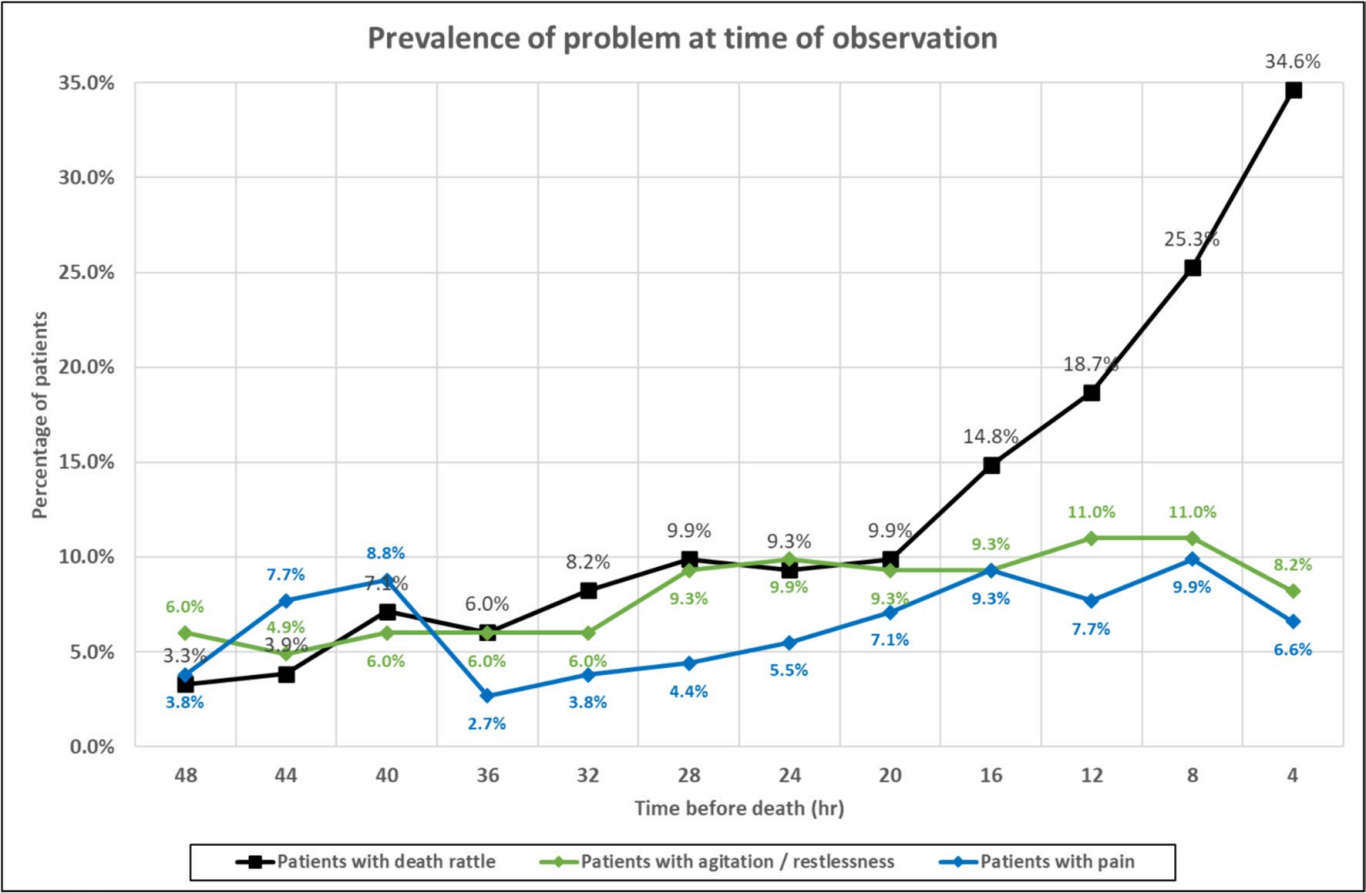
Prevalence of audible upper airway secretions (“death rattle”), restlessness / agitation, and pain in last 48 h of life

The presence of audible secretions in the last 48 h of life was associated with the presence of restlessness / agitation (as measured on m-RASS) at the 4 hourly observations: χ^2^ (1, *n* = 972) = 39.7738, *p* < 0.00001); odds ratio = 3.6188 (95% CI: 2.3801–5.5022). However, at most (79.6%) time points, patients with audible secretions were not deemed restless / agitated. Similarly, the presence of audible secretions in the last 48 h of life was associated with the presence of pain at the 4 hourly nursing observations: χ^2^ (1, *n* = 974) = 20. 461, *p* < 0.00001); odds ratio = 3.0306 (95% CI: 1.8352–5.0046). Again, however, at most (87.3%) time points, patients with audible secretions were not deemed in pain. The prevalence of audible secretions, restlessness / agitation, and pain during the last 48 h of life is shown in Fig. 2.

Forty-four patients received a regular anticholinergic (subcutaneous infusion) for audible secretions: these included 25 patients that had audible secretions at death, and 19 patients that did not have this problem (including five patients that had never exhibited this problem). Ninety patients received an as-required anticholinergic (subcutaneous injection) for audible secretions: these included 48 patients that had audible secretions at death. The most commonly prescribed anticholinergic was glycopyrronium bromide (62%), with the other drugs utilised being hyoscine butylbromide (29%), and hyoscine hydrobromide (9%).

## Discussion

The label “death rattle” reflects that this problem is a frequent precursor (“harbinger) to death [17, 18]. This study reaffirms this point, and demonstrates that the 4 hourly prevalence of audible upper airway secretions increases significantly over the last hours of life. Although several authors have suggested the latter [19], there is minimal corroborating evidence within the literature (i.e. prospective research studies) [20]. Hui et al. assessed “clinical signs of impending death” every 12 h, and reported an increase in the prevalence of audible secretions in the last 48 h of life [20]. This study also shows that audible secretions can occur at earlier timepoints, when they can disappear (with or without treatment), can reappear, and even involve multiple distinct episodes [21].

This study found an association between male sex and the development of audible secretions. It is difficult to explain this finding, especially given the lack of an association with male-associated cancer diagnoses or comorbidities. Certain other studies have reported a similar finding [22], but a systematic review of the literature noted that most studies reported no effect of sex (with one study reporting an association with female sex) [23]. In terms of “risk factors”, the latter systematic review concluded that “there was a weak but consistent association between brain and/or lung metastases *(but otherwise nothing)* and the development of death rattle” [23].

Importantly, this study found no association between CAH and the development of audible secretions. Other studies have similarly reported no association between hydration status and the development of audible secretions [24], or indeed the use of CAH and the development of audible secretions [25]. Importantly, the CHELsea II (definitive) study will assess this association in a much larger number of patients [26]. On the basis of the proposed aetiology of audible secretions, it is difficult to conceive how CAH could (negatively) impact the development of audible secretions [11].

Surprisingly, this study found a statistically significant association between the presence restlessness / agitation (and pain) and the presence of audible secretions. We found no supporting evidence within the literature, and there are multiple references to the fact that patients with audible secretions do not generally appear to be distressed (suggesting an absence of agitation) [6]. Additionally, Campbell et al. reported that audible secretions were not associated with “respiratory distress” [27]. Reassuringly, this study noted that most patients with audible secretions were not restless / agitated (or in pain). It is unclear why such an association would exist, and this will be further examined in the ongoing (larger) CHELsea II trial [26].

In terms of management, the “usual” interventions for audible secretions involve repositioning of the patient, oropharyngeal suctioning, reassurance for the relatives (around the absence of patient distress), and use of anticholinergic medication [6], although other strategies have been suggested to manage this condition (i.e. reduction in rate of respiration, bronchodilation) [11]. The relevant Cochrane systematic review concluded that “there was no evidence to show that any intervention, be it pharmacological or non-pharmacological, was superior to placebo in the treatment of noisy breathing” [5]. However, the data from this study, and clinical experience, suggests that many patients with audible secretions do improve following treatment with anticholinergic medication, although whether this is due to the medication or natural history remains undetermined.

The main strengths of this study are the trial design (i.e. multicentre randomised controlled trial), and the routine / regular assessment of audible secretions. The main limitations of this study are the sample size (i.e. 200 participants), and the subjective assessment of audible secretions. Thus, the assessment was clinical in nature, and necessitated differentiation between “death rattle” and other potential causes of “noisy breathing”. Importantly, there is no method of differentiating between the different types of audible upper airway secretions.

## Conclusion

Audible secretions (“death rattle”) are common in cancer patients at the end-of-life, but their natural history is extremely variable, with some patients experiencing multiple episodes during the terminal phase (although not necessarily experiencing an episode at the time of death).
